# Zoom-in Dermoscopy for Facial Tumors

**DOI:** 10.3390/diagnostics15030324

**Published:** 2025-01-30

**Authors:** Martina D’Onghia, Francesca Falcinelli, Lorenzo Barbarossa, Alberto Pinto, Alessandra Cartocci, Linda Tognetti, Giovanni Rubegni, Anastasia Batsikosta, Pietro Rubegni, Elisa Cinotti

**Affiliations:** 1 Dermatology Unit, Department of Medical, Surgical and Neurological Sciences, University of Siena, 51300 Siena, Italy; f.falcinelli@student.unisi.it (F.F.); lorenzobarbarossa1993@gmail.com (L.B.); alberto.pinto@student.unisi.it (A.P.); alessandra.cartocci@dbm.unisi.it (A.C.); linda.tognetti@dbm.unisi.it (L.T.); pietro.rubegni@gmail.com (P.R.); elisa.cinotti@unisi.it (E.C.); 2 Department of Clinical Medicine and Immunological Sciences, Section of Ophthalmology, University of Siena, 51300 Siena, Italy; giovannirubegni@gmail.com; 3 Pathological Anatomy Section, University of Siena, 51300 Siena, Italy; natasha.batsikosta@gmail.com

**Keywords:** dermoscopy, high magnification, FAV, melanocyte, lentigo maligna, melanoma

## Abstract

**Background/Objectives**: Facial lesions, including lentigo maligna and lentigo maligna melanoma (LM/LMM), both malignant, present significant diagnostic challenges due to their clinical similarity to benign conditions. Although standard dermoscopy is a well-established tool for diagnosis, its inability to reveal cellular-level details highlights the necessity of new magnified techniques. This study aimed to assess the role of standard dermoscopy, high-magnification dermoscopy, and fluorescence-advanced videodermatoscopy (FAV) in diagnosing LM/LMM and differentiating them from benign facial lesions. **Methods**: This retrospective, observational, multicenter study evaluated 85 patients with facial skin lesions (including LM, LMM, basal-cell carcinoma, solar lentigo, seborrheic keratosis, actinic keratosis, and nevi) who underwent dermatological examination for skin tumor screening. Standard dermoscopy at 30× magnification (D30), high-magnification dermoscopy at 150× magnification (D150), and FAV examination were performed. Dermoscopic images were retrospectively evaluated for the presence of fifteen 30× and twenty-one 150× dermoscopic features, and their frequency was calculated. To compare D30 with D150 and D150 with FAV, the Gwet AC1 concordance index and the correct classification rate (CCR) were estimated. **Results**: Among 85 facial lesions analyzed, LM/LMM exhibited distinctive dermoscopic features at D30, including a blue–white veil (38.9% vs. 1.7%, *p* < 0.001), regression structures (55.6% vs. 21.7%, *p* = 0.013), irregular dots or globules (50.0% vs. 10%, *p* = 0.001), angulated lines (72.2% vs. 6.7%, *p* < 0.001), an annular granular pattern (61.1% vs. 20%, *p* = 0.002), asymmetrical pigmented follicular openings (100.0% vs. 21.7%; *p* < 0.001), and follicular obliteration (27.8% vs. 3.3%). At D150, roundish melanocytes (87.5% vs. 18.2%, *p* < 0.001) and melanophages (43.8% vs. 14.5%, *p* = 0.019) were predominant. FAV examination identified large dendritic cells, isolated melanocytes, and free melanin in LM/LMM (all *p* < 0.001) with high concordance to D150. **Conclusions**: Integrating D30, D150, and FAV into clinical practice may enhance diagnostic precision for facial lesions by combining macroscopic and cellular insights, thereby reducing unnecessary biopsies. However, future studies are essential to confirm these results.

## 1. Introduction

Diagnosing pigmented skin lesions on the face is challenging for dermatologists, mainly because of the clinical overlap between benign and malignant conditions [1]. In fact, typical lesions of photo-aged skin, such as solar lentigo (SL), seborrheic keratosis (SK), and actinic keratosis (AK), especially in pigmented forms, frequently mimic melanocytic lesions, complicating diagnosis [2]. Lentigo maligna (LM) and lentigo maligna melanoma (LMM) are slow-growing tumors that primarily affect white elderly individuals, typically developing on chronically sun-exposed areas of the head and neck. LM refers to in situ lesions, while invasive forms are classified as LMM [3]. These tumors usually present as irregularly pigmented macules and can often grow to a significant size before being detected [3].

Standard dermoscopy with magnifications up to 30× (D30) is a widely recognized tool that enhances diagnostic accuracy for skin tumors. Indeed, the use of criteria based on distinct patterns and structures in both melanocytic and non-melanocytic lesions significantly improves diagnostic precision [4]. This technique is especially valuable for reducing unnecessary biopsies, especially in the facial areas.

The unique characteristics of facial skin, such as thin epidermis and high exposure to sunlight, contribute to the diverse dermoscopic patterns observed. Benign lesions, including SK or SL, often display features such as milia-like cysts or comedo-like openings, whereas AK commonly presents with a pseudonetwork pattern and a strawberry-like appearance [5]. In contrast, dermoscopic criteria to identify LM and LMM include dots, gray globules, asymmetric follicular openings, rhomboidal structures, and pigmentation surrounding hair follicles up to follicular obliteration [3,6]. Despite these diagnostic clues, differentiating between benign and malignant lesions on the face is still challenging due to overlapping features and subtle presentations. This highlights the need for further advancements in dermoscopic techniques, such as magnified dermoscopy, to improve the diagnostic accuracy of those lesions.

The advent of high-magnification videodermoscopy with magnification up to 150× (D150) has significantly enhanced diagnostic capabilities by providing unprecedented detail, enabling the visualization and differentiation of individual pigmented cells, such as keratinocytes and melanocytes, particularly in cases where standard magnification is insufficient to identify critical diagnostic features, thereby offering a more precise evaluation of complex lesions [7,8].

Fluorescence-advanced videodermoscopy (FAV) has also emerged as a new technique for the non-invasive, rapid, and dynamic examination of superficial skin structures at cellular-level resolution [9]. By using the fluorescence emitted by endogenous molecules upon absorption at specific wavelengths, FAV enables in vivo imaging through direct application to the skin. This method facilitates real-time scanning across various skin depths, presenting grayscale images in which the fluorescence intensity ranges from black to white [9]. Although previous studies have highlighted the potential of FAV for improving the diagnostic accuracy of flat pigmented facial lesions, research on this emerging technique remains limited, with only a few studies conducted to date [10]. Moreover, comparative studies between FAV and high-magnification dermoscopy are scarce, underscoring the need for more comprehensive research to clarify their respective roles in dermatological diagnostics.

Against this background, we evaluated the diagnostic value of high-magnification dermoscopy for pigmented facial lesions by comparing its findings with those of traditional dermoscopy. In addition, we investigated whether higher magnification could uncover previously undetectable structures, thereby aiding in the differentiation of LM, LMM, and benign facial lesions. Finally, FAV was used to analyze facial lesions, and its findings were compared with those of high-magnification dermoscopy to further explore its diagnostic utility.

## 2. Materials and Methods

We conducted an observational and retrospective study between November 2023 and June 2024 on non-consecutive patients who underwent dermatological examination for skin tumor screening at the Dermatology Department of the University Hospital of Siena, Italy.

This study enrolled patients presenting with facial lesions that, based on the evaluation of an expert dermatologist (E.C.), required either removal or 12-month follow-up due to atypical clinical or dermoscopic features. The following lesions were included: LM, LMM, basal-cell carcinoma (BCC), SL, SK, AK, pigmented AK (PAK), lichenoid keratosis (LK), and nevi.

Double-magnification, polarized light videodermatoscopy images were captured at 30× and 150× magnification using Horus System HS600^®^ (Adamo Srl., Trapani, Italy). Specialists in skin imaging (E.C. and F.F.), acquired at least 5 images for each lesion. In addition, in some cases, advanced fluorescence videodermatoscopy (Horus^®^ handled probe, Adamo Srl.) at 500× magnification was performed.

Images at 150× magnification were captured from the most characteristic areas identified at 30× magnification by rotating the ring on the videodermoscope probe, allowing for real-time zooming into the details of the 30× images. FAV images were obtained using a separate probe connected to the same computer system as the 30×/150× probe. The field of view has a diameter of 8 mm, 1.7 mm, and 0.34 mm for 30×, 150×, and FAV, respectively.

According to the current literature, the following dermoscopic patterns were assessed for 30× evaluation: blue–white veil, atypical vascular pattern, regression structures, irregular blotches, irregular dots or globules, white and wide follicular opening, reticular or parallel brown lines, sharply demarcated borders, milia-like cysts or comedo-like openings, erythematous pseudonetwork pattern, pseudonetwork pattern, angulated lines (which include polygons or rhomboids or a zig-zag pattern), annular granular pattern or gray circles, asymmetrical pigmented follicular openings, and follicular obliteration [11,12].

For high magnification, we considered the following variables identified in our previous studies: the presence of pigmented cells (keratinocytes, roundish or dendritic melanocytes, melanophages) and their features (distribution, size, or shape regularity); dots (round pigmented areas smaller than a cell); nests of cells (roundish pigmented areas formed by >1 cell); structureless areas that do not follow the DEJ architecture; vessels and their shape (linear, glomerular, arborizing, dilated inside the dermal papilla, or irregular); hyperkeratotic, roundish, concentric structures; pigmented network delimiting well-defined dermal papillae (edged papillae) or undefined dermal papillae (nonedged papillae, not delimited by single cells); and keratin plug inside hair follicle, multiple shades of brown, out-of-focus purple-bluish structureless area, and folliculotrophism [13].

Finally, we identified the following terms to describe the parameters observed with FAV: small, pigmented cells, large isolated cells with clearly visible sharp borders, large isolated dendritic cells, and free melanin [10].

For all selected cases, a correlation between 30× and 150× and between 150× and FAV features was performed. The images were evaluated by a group of three expert dermatologists (L.B., M.D., and F.F.), who were blinded to the histological diagnoses.

Descriptive statistics included the mean and standard deviation (SD) for quantitative variables, whereas frequency and percentage were reported for categorical variables. To compare D30 with D150 and D150 with FAV, the Gwet AC1 concordance index and the correct classification rate (CCR) were estimated. *p* < 0.05 was considered statistically significant. All analyses were performed using R software version 4.1.0 (R Foundation for Statistical 100 Computing, Vienna, Austria).

## 3. Results

A total of 85 patients were included in this study, with a mean age (SD) at diagnosis of 64.24 (13.04) years (Table 1). Most of the patients were female (55.3%). All pigmented lesions were located on the face, with the cheeks being the most affected site (35.3%), followed by the nose (23.6%), forehead (16.5%), and scalp (13%). Less frequent locations included the eyelids, which were involved in five cases, the neck in three cases, and the ears and chin, with each contributing one case. Overall, histological examination was performed in 47 patients (55.3%). Among benign lesions, LS was the most frequently identified subtype, observed in 24 cases (28.2%), followed by SK (10.6%), PAK (9.4%), nevi (9.4%), and AK (8.2%). LM and LMM were identified in 14 and 4 patients, respectively.

### 3.1. Dermoscopy at 30× Magnification

The D30 features are listed in Table 2.

Concerning malignant benchmarks, the blue–white veil (38.9% vs. 1.7%, *p* < 0.001), regression structures (55.6% vs. 21.7%, *p* = 0.013), irregular dots or globules (50.0% vs. 10%, *p* = 0.001), angulated lines (72.2% vs. 6.7%, *p* < 0.001), annular granular pattern (61.1% vs. 20%, *p* = 0.002), asymmetrical pigmented follicular openings (100.0% vs. 21.7%; *p* < 0.001), and follicular obliteration (27.8% vs. 3.3%) were more commonly observed in LM or LMM lesions compared to other skin lesions. As for white and wide follicular openings (76.7% vs. 27.8%, <0.001), reticular or parallel brown lines (38.3% vs. 0%, *p* = 0.005), and pseudonetwork pattern (56.7% vs. 0%, *p* < 0.001), these patterns were primarily observed in non-LM/LMM lesions.

### 3.2. Dermoscopy at 150× Magnification

The D150 features are presented in Table 3.

**Figure 1:**
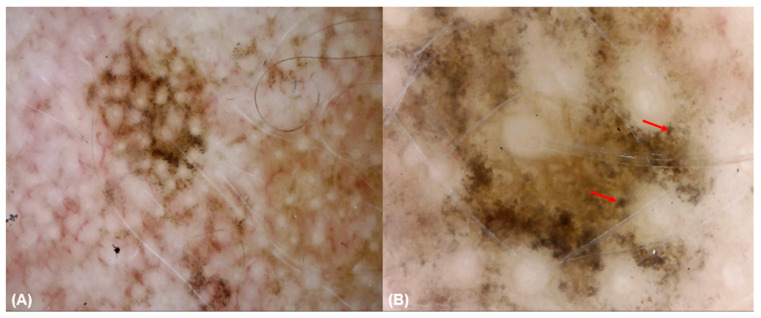
A lentigo maligna at 30× (**A**) and 150× (**B**) magnification. The 30× magnification dermoscopy (**A**) reveals asymmetrical pigmented follicular openings and annular granular pattern. The 150× magnification dermoscopy (**B**) shows a network without well-defined dermal papillae and the presence of large, irregular (in size and shape) roundish (red arrow) cells, corresponding to melanocytes.

Keratinocytes (100% vs. 71.4%, *p* = 0.005) and regular cell distribution (65.5% vs. 14.3%, *p* = 0.038) were significantly more common in other facial lesions than BCC. Roundish melanocytes were more indicative of LM/LMM, compared to non-LM/LMM lesions (*p* < 0.001). Similarly, dendritic melanocytes (43.8% vs. 14.5%, *p* = 0.019), melanophages (43.8% vs. 14.5%, *p* = 0.019), pigmentation without edged papillae (93.8% vs. 40%, *p* < 0.001), multiple shades of brown (81.2% vs. 23.6%, *p* < 0.001), and out-of-focus purple-bluish structureless areas (37.5% vs. 5.5%, *p* = 0.001) were present in LM/LMM lesions compared to other facial lesions (Figure 1).

**Figure 2:**
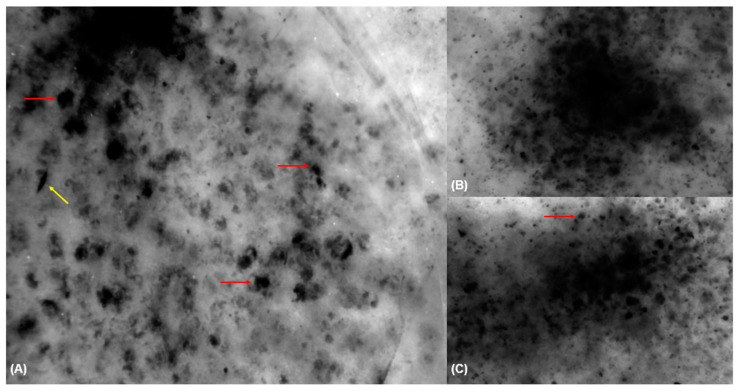
Fluorescence-advanced videodermoscopy of a lentigo maligna (**A**–**C**). FAV (**A**,**C**) shows large isolated cells with clearly visible sharp borders (red arrow) and (**C**) isolated dendritic cells (yellow arrow) corresponding to malignant melanocytes.

Conversely, pigmentation with edged papillae was more indicative of non-LM/LMM than LM/LMM lesions (50.9% vs. 18.8%, *p* = 0.005) (Figure 2). Finally, vessel shape showed significant differences, being predominant in BCC lesions (71.4%), compared to other lesions (27.3% and 25%, non-LM/LMM and LM/LMM, respectively).

### 3.3. Dermoscopy at 30× Compared with 150× Magnification

The agreement between D30× and D150× magnifications was generally moderate to good across all parameters evaluated (all *p* < 0.001) (Table 4). However, significant differences in the prevalence of features were observed between D150× and D30×. These included roundish or dendritic melanocytes and angulated lines (41% vs. 20%, *p* = 0.006), roundish or dendritic melanocytes and follicular obliteration (41% vs. 8.2%, *p* < 0.001), melanophages and follicular obliteration (19.2% vs. 8.2%, *p* = 0.010), cell irregularity in morphology and follicular obliteration (35.9% vs. 8.2%, *p* < 0.001), and folliculotropism with asymmetrical pigmented follicular openings (15.4% vs. 8.2%, *p* = 0.001).

### 3.4. Fluorescence-Advanced Videodermatoscopy Imaging

**Figure 3:**
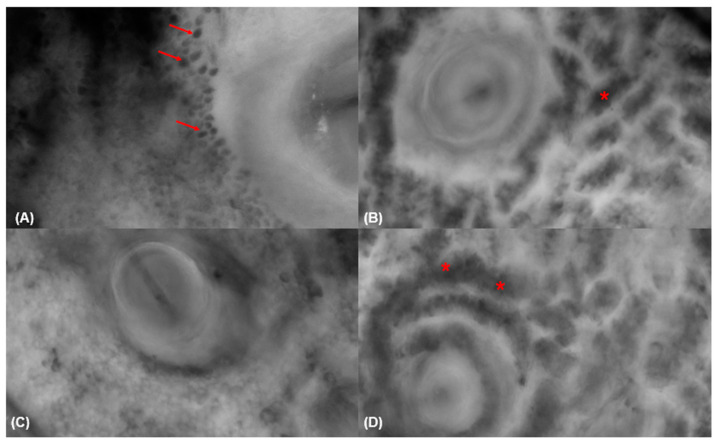
Fluorescence-advanced videodermoscopy of a solar lentigo (**A**–**D**). FAV (**A**) shows interfollicular arrangement of the pigmented cell with follicle sparing corresponding to keratinocytes (red arrow) and (**B**–**D**) pseudo-tubular formations (red asterisks).

Large isolated cells with clearly visible sharp borders (93.8% vs. 2.4%, *p* < 0.001), large isolated dendritic cells (68.8% vs. 9.5%, *p* < 0.001), and free melanin (93.8% vs. 8.1%, *p* < 0.01) were mainly observed in skin lesions diagnosed as LM/LMM lesions compared to other lesions (Table 5) (Figure 2 and Figure 3).

Overall, the prevalence of D150 and FAV features assumed was consistent across the two techniques (all *p* > 0.05), with a strong concordance between the two methods (*p* < 0.001), although a slightly lower agreement was noted for dendritic melanocytes and large isolated cells (Gwet AC1: 0.50) (Table 6).

## 4. Discussion

This study provides a comprehensive evaluation of standard dermoscopy, high-magnification dermoscopy, and FAV in diagnosing pigmented lesions, emphasizing their complementary contributions to enhancing diagnostic accuracy for LM/LMM and their benign mimics.

As expected, conventional dermoscopy at D30 magnification is a reliable tool for detecting the distinctive features of both malignant and benign lesions, which is consistent with previous findings in the existing literature [14]. According to our results, LM/LMM lesions exhibited the well-documented dermoscopic criteria for malignancy compared to benign lesions, including blue–white veil (38.9% vs. 1.7%, *p* < 0.001), regression structures (55.6% vs. 21.7%, *p* = 0.013), irregular dots or globules (50.0% vs. 10%, *p* = 0.001), angulated lines (72.2% vs. 6.7%, *p* < 0.001), annular granular pattern (61.1% vs. 20%, *p* = 0.002), asymmetrical pigmented follicular openings (100.0% vs. 21.7%; *p* < 0.001) and follicular obliteration (27.8% vs. 3.3%). On the other hand, benign benchmarks, including white and wide follicular openings (76.7% vs. 27.8%, <0.001), reticular or parallel brown lines (38.3% vs. 0%, *p* = 0.005), and pseudonetwork pattern (56.7% vs. 0%, *p* < 0.001), were mainly present in non-LM/LMM lesions.

**Figure 4:**
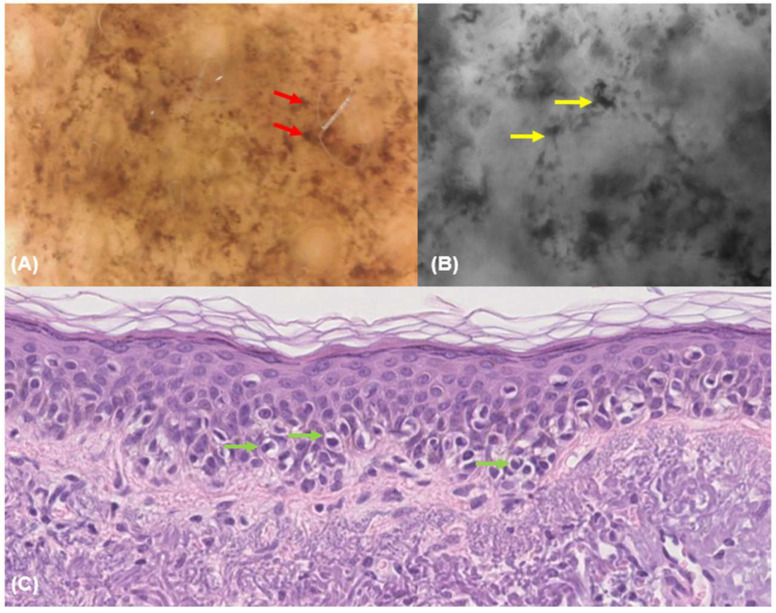
Dermoscopy at 150× magnification (**A**), fluorescence-advanced videodermoscopy, (**B**) and histological image (**C**) of a lentigo maligna melanoma. Dermoscopy at 150× magnification (**A**) reveals melanocytes and melanocytic invasion of a hair follicle (red arrow). FAV (**B**) reveals pigmented cells that are irregular in shape and size and correspond to malignant melanocytes (yellow arrow). Histological image (**C**) shows a proliferation of intraepidermal melanocytes overlying solar elastosis (green arrow). Hematoxylin and eosin; original magnification 20×.

**Figure 5:**
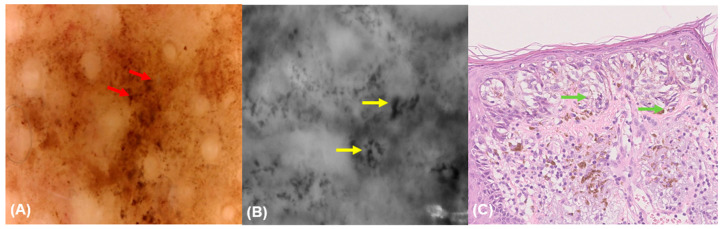
Dermoscopy at 150× magnification (**A**), fluorescence-advanced videodermoscopy, (**B**) and histological image (**C**) of a lentigo maligna melanoma. Dermoscopy at 150× magnification (**A**) reveals melanocytes and melanocytic invasion of a hair follicle (red arrow). FAV (**B**) reveals pigmented cells that are irregular in shape and size and correspond to malignant melanocytes (yellow arrow). Histological image (**C**) shows a proliferation of intraepidermal melanocytes with irregular distribution of nests (green arrow). Hematoxylin and eosin; original magnification 20×.

Although conventional dermoscopy is an essential tool for diagnosing LM/LMM, its ability to reveal cellular and subcellular details remains inherently limited. This limitation highlights the value of complementary magnified techniques, which enable cellular-level visualization and help bridge the gap between traditional imaging and histopathology [15]. These advanced methods offer a promising opportunity to correlate dermoscopic findings with histopathological findings, thereby enhancing diagnostic precision (Figure 4 and Figure 5).

**Figure 6:**
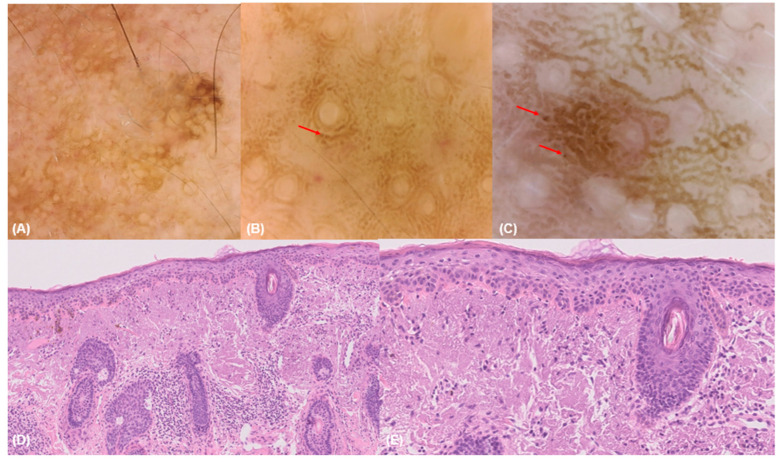
Dermoscopy at 30× (**A**) and 150× (**B**,**C**) magnification and histological images (**D**,**E**) of a solar lentigo. Dermoscopy at 30× magnification (**A**) reveals white and wide follicular openings. Dermoscopy at 150× magnification (**B**,**C**) shows round keratinocytes with a pseudo-tubular distribution (red arrows) around the follicular openings. Histological images (**D**,**E**) reveal atypia of basal keratinocytes with loss of polarization and intense solar elastosis. Hematoxylin and eosin; original magnification 10× (**D**), and 20× (**E**).

In this context, the Horus videodermoscope allows for real-time zoom into a selected area of a conventional dermoscopy image at 30× magnification, providing highly magnified images with cytological detail. This approach mirrors the histopathological process, where an initial examination is conducted at a lower magnification, followed by a closer inspection of specific areas at higher magnification to observe finer details (Figure 6).

**Figure 7:**
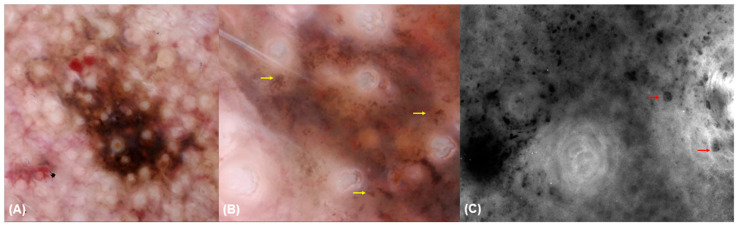
Dermoscopy at 30× (**A**) and 150× (**B**) magnification and fluorescence-advanced videodermoscopy of a lentigo maligna. Dermoscopy at 30× magnification (**A**) reveals asymmetrical pigmented follicular openings and follicular obliteration. Dermoscopy at 150× magnification (**B**) shows melanocytes and melanocytic invasion of a hair follicle (yellow arrow). FAV (**C**) shows pigmented cells that are irregular in shape and size and correspond to malignant melanocytes (red arrow).

Consistent with previous studies on the use of magnified dermoscopy for evaluating facial lesions [16], our findings demonstrated that features such as roundish melanocytes (87.5% vs. 18.2%, *p* < 0.001), dendritic melanocytes (43.8% vs. 14.5%, *p* = 0.019), melanophages (43.8% vs. 14.5%, *p* = 0.019), pigmentation without edged papillae (93.8% vs. 40%, *p* < 0.001), multiple shades of brown (81.2% vs. 23.6%, *p* < 0.001), and out-of-focus purple-bluish structureless areas (37.5% vs. 5.5%, *p* = 0.001) observed at D150× were significantly more common in LM/LMM compared with other lesions (Figure 7).

Notably, a pilot study by Cinotti et al. [17] demonstrated that images acquired with a videodermoscope offering similar magnification (D400 or super-high magnification dermoscopy, Fotofinder Medicam 1000, Bad Birnbach, Germany) can assist in the non-invasive diagnosis of MM by visualizing individual pigmented cells [17]. However, unlike the Fotofinder system, the Horus device offers a significant advantage because operators can acquire magnified dermoscopy images simply by rotating a ring on the probe to zoom in on the area of interest without changing the final lens.

Regarding vascular structures, our analysis revealed significant differences at D150, with vessels being more prevalent in BCC lesions (71.4%) compared with AK/LS (27.3%) and LM/LMM (25%). Notably, BCC lesions predominantly exhibited arborizing vessels (85.7%). The dermoscopic diagnosis of BCC largely depends on the absence of a pigment network and the identification of one or more of six key dermoscopic criteria, including arborizing telangiectasia [18]. In this context, magnified dermoscopy has the potential to visualize vascular structures with unprecedented clarity, and it is particularly valuable when characteristic pigmented features are absent in standard dermoscopy.

As shown in Table 4, our comparative analysis of conventional and magnified dermoscopy parameters revealed moderate to good agreement for most of the evaluated features, with concordance levels ranging from 0.432 to 0.871. These findings suggest that the two techniques are not entirely concordant. A distinctive, albeit not entirely specific, feature of LM/LMM is the invasion of follicular structures, which can be indirectly observed as pigmentation surrounding hair follicles [19]. At the cellular level, this phenomenon is likely explained by the migration of melanocytes and melanophages into follicular structures as the lesion progresses [19]. These cellular elements, as previously mentioned, can be directly visualized using D150 dermoscopy.

In our analysis, the differences in the prevalence of analyzed parameters, such as melanophages at D150 (19.2%) and follicular obliteration at D30 (8.2%), confirm that the two techniques, due to their differing levels of magnification, provide different details of the lesion. However, the high level of concordance (Gwet AC1 0.749, *p* < 0.001) indicates that in cases where follicular obliteration was identified at 30× magnification, melanophages were consistently visible at 150× magnification. This synergistic relationship enhances diagnostic specificity for facial lesions, highlighting the added value of integrating both approaches and emphasizing the importance of employing both methods for comprehensive evaluation. Furthermore, these findings underscore the utility of high-magnification imaging for identifying diagnostic features that remain undetectable in conventional dermoscopy alone.

Similarly to D150, FAV is an innovative non-invasive imaging technique that combines videodermoscopy with information derived from the autofluorescence of skin molecules such as hemoglobin and melanin, enabling the visualization of pigmented keratinocytes and melanocytes [9]. Recently, Scrafì et al. [10] analyzed 21 consecutive suspected facial lesions, including LM, LMM, SL, SK, and PAK, and concluded that FAV features offer an enhanced diagnostic approach for differentiating flat pigmented facial lesions.

Our results further confirmed the diagnostic potential of FAV for identifying malignant features. Specifically, we observed that large, isolated cells with sharp, well-defined borders, large dendritic cells, and free melanin were significantly more prevalent in LM/LMM than in other lesions (all *p* < 0.001). Notably, the high concordance between FAV and D150 in our study suggested that FAV could serve as a reliable alternative to D150 in clinical practice (Table 6).

The current study highlights the importance of integrating traditional and magnified dermoscopy techniques to achieve a more comprehensive understanding of facial lesions.

The ability of D150 and FAV to reveal cellular details that are not detectable with D30 may offer valuable insights into the histopathological correlates of dermoscopic features, ultimately enhancing diagnostic accuracy and informing clinical decision making. Notably, the features observed at 30× magnification often differ from those seen at 150×, with each level offering unique advantages. While 150× magnification enables the visualization of fine cytological details, 30× magnification provides a broader perspective, allowing for a clearer assessment of pigmentation patterns of the skin. Moreover, the observed concordance between the modalities underscores the potential of these techniques to complement each other, providing unique diagnostic information.

However, our study has some limitations. First, the acquisition of D150 and FAV images was operator-dependent, potentially introducing variability in the selection of areas for examination. Second, the image interpretation was retrospective. Finally, this study did not assess correlations between histopathological images.

## 5. Conclusions

Integrating D30, D150, and FAV into routine clinical practice may improve the diagnosis of facial lesions and minimize unnecessary biopsies. Moreover, a deeper understanding of these techniques could bridge the gap between dermoscopy and histology, advancing the field of dermoscopic imaging. Nevertheless, further research is required in this field.

## Figures and Tables

**Table 1:** Clinical and histological characteristics of facial lesions in our study population.

	Overall
	n = 85
	n (%)
Female	47 (55.3)
Age at diagnosis, mean (SD)	64.24 (13.04)
Specific areas involved	
Cheeks	30 (35.3)
Nose	20 (23.6)
Forehead	14 (16.5)
Scalp	11 (13)
Eyelids	5 (5.9)
Neck	3 (3.5)
Ears	1 (1.2)
Chin	1(1.2)
Histological examination	47 (55.3)
Lesion subtypes	
SL	24 (28.2)
LM	14 (16.5)
SK	9 (10.6)
Naevus	8 (9.4)
PAK	8 (9.4)
AK	7 (8.2)
BCC	7 (8.2)
KL	4 (4.7)
LMM	4 (4.7)

Legend: AK, actinic keratosis; BCC, basal-cell carcinoma; KL, lichenoid keratosis; LM, lentigo maligna; LMM, lentigo maligna melanoma; SL, solar lentigo; PAK, pigmented actinic keratosis; SK, seborrheic keratosis.

**Table 2:** Dermoscopy features of facial lesions at 30× magnification.

	Other * N = 60 n (%)	LM or LMM N = 18 n (%)	*p*
White and wide follicular opening	46 (76.7)	5 (27.8)	<0.001
Reticular or parallel brown lines	23 (38.3)	0 (0.0)	0.005
Sharply demarcated borders	30 (50.0)	5 (27.8)	0.164
Milia-like cysts or comedo-like openings	4 (6.7)	0 (0.0)	0.606
Blue–white veil	1 (1.7)	7 (38.9)	<0.001
Atypical vascular pattern	1 (1.7)	1 (5.6)	0.948
Regression structures	13 (21.7)	10 (55.6)	0.013
Blotches irregularly distributed	2 (3.3)	1 (5.6)	1.000
Irregular dots or globules	6 (10.0)	9 (50.0)	0.001
Erythematous pseudonetwork pattern	8 (13.3)	4 (22.2)	0.586
Pseudonetwork pattern	34 (56.7)	0 (0.0)	<0.001
Angulated lines	4 (6.7)	13 (72.2)	<0.001
Annular granular pattern or gray circles	12 (20.0)	11 (61.1)	0.002
Asymmetrical pigmented follicular openings	13 (21.7)	18 (100.0)	<0.001
Follicular obliteration	2 (3.3)	5 (27.8)	0.007

Legend: * this group included actinic keratosis; lichenoid keratosis; solar lentigo; pigmented actinic keratosis, and seborrheic keratosis. LM, lentigo maligna; LMM, lentigo maligna melanoma.

**Table 3:** Dermoscopy features of facial lesions at 150× magnification.

	BCC N = 7 n (%)	Other * N = 55 n (%)	LM or LMM N = 16 n (%)	*p*
Cell presence	6 (85.7)	55 (100.0)	16 (100.0)	0.090
Cell type				
Keratinocytes	5 (71.4)	55 (100.0)	15 (93.8)	0.005 a
Roundish melanocytes	1 (14.3)	10 (18.2)	14 (87.5)	<0.001 b,c
Dendritic melanocytes	-	8 (14.5)	7 (43.8)	0.019
Melanophages	-	8 (14.5)	7 (43.8)	0.019
Cell irregularity in shape and size	3 (42.9)	12 (21.8)	13 (81.2)	<0.001 c
Cell distribution				
Regular cell distribution	1 (14.3)	36 (65.5)	9 (56.2)	0.038 a
Roundish nests	1 (14.3)	5 (9.1)	0 (0.0)	0.362
Dots	3 (42.9)	12 (21.8)	7 (43.8)	0.132
Structureless area that do not follow DEJ architecture	1 (14.3)	7 (12.7)	4 (25.0)	0.458
Vessels	5 (71.4)	15 (27.3)	4 (25.0)	0.065
Vessel shape				<0.001
No vessels	1 (14.3)	41 (74.5)	12 (75.0)	
Linear	-	9 (16.4)	4 (25.0)	
Arborizing	6 (85.7)	3 (5.5)	-	
Polymorphous	-	2 (3.6)	-	
Out of focus purple-bluish, structureless areas	3 (42.9)	3 (5.5)	6 (37.5)	0.001 a,c
Multiple shades of brown	2 (28.6)	13 (23.6)	13 (81.2)	<0.001 b,c
Hyperkeratotic roundish concentric areas	-	8 (14.5)	1 (6.2)	0.608
Pigmentation with edged papillae	-	28 (50.9)	3 (18.8)	0.005 c
Pigmentation without edged papillae	5 (71.4)	22 (40.0)	15 (93.8)	<0.001 c
Keratin plugs inside hair follicles	1 (14.3)	10 (18.2)	-	0.172

Legend: * this group included actinic keratosis, lichenoid keratosis, solar lentigo, pigmented actinic keratosis, and seborrheic keratosis (SK). LM, lentigo maligna; LMM, lentigo maligna melanoma. (a) BCC significantly different from others, (b) BCC significantly different from LM/LMM, (c) others significantly different from LM/LMM.

**Table 4:** Relative frequencies and agreement between D150 and D30 features.

		Agreement		Accuracy	Prevalence		
150×	30×	Gwet AC1	*p*		Prevalence 150×	Prevalence 30×	*p*
Roundish or dendritic melanocytes	Angulated lines	0.579	<0.001	75.6	41.0%	20.0	0.006
	Annular granular pattern or gray circles	0.531	<0.001	74.4%	41.0	27.1	0.085
	Asymmetrical pigmented follicular openings	0.481	<0.001	73.1	41.0	38.8	0.899
	Follicular obliteration	0.432	<0.001	64.1	41.0	8.2	<0.001
Melanophages	Angulated lines	0.591	<0.001	71.8	19.2	20.0	1.000
	Annular granular pattern or gray circles	0.658	<0.001	78.2	19.2	27.1	0.319
	Asymmetrical pigmented follicular openings	0.385	<0.001	64.1	19.2	38.8	0.010
	Follicular obliteration	0.749	<0.001	80.7	19.2	8.2	0.068
Cell irregularity in shape and size	Angulated lines	0.552	<0.001	73.1	35.9	20.0	0.036
	Annular granular pattern/gray circles	0.591	<0.001	76.9	35.9	27.1	0.295
	Asymmetrical pigmented follicular openings	0.492	<0.001	73.0	35.9	38.8	0.823
	Follicular obliteration	0.533	<0.001	69.2	35.9	8.2	<0.001
Pigmentation with edged papillae	Pseudonetwork pattern	0.603	<0.001	79.5	39.7	40.0	1.00
Polymorphus vessels	Atypical vascular pattern	0.854	<0.001	87.2	2.6	9.4	0.135
Folliculotropism	Angulated lines	0.802	<0.001	85.9	15.4	20.0	0.572
	Annular granular pattern or gray circles	0.650	<0.001	76.9	15.4	27.1	0.104
	Asymmetrical pigmented follicular openings	0.552	<0.001	73.1	15.4	38.8	0.001
	Follicular obliteration	0.871	<0.001	89.7	15.4	8.2	0.239

**Table 5:** Fluorescence-advanced videodermatoscopy features of facial lesions.

	Other * N = 42 n (%)	LM/LMM N = 16 n (%)	*p*
Small pigmented cells with sharp borders	38 (90.5)	16 (100.0)	0.484
Large isolated cells with clearly visible sharp borders	1 (2.4)	15 (93.8)	<0.001
Large isolated dendritic cells	4 (9.5)	11 (68.8)	<0.001
Free melanin	16 (8.1)	15 (93.8)	<0.001

Legend: * this group included actinic keratosis, basal-cell carcinoma, lichenoid keratosis, solar lentigo, pigmented actinic keratosis, and seborrheic keratosis.

**Table 6:** Relative frequencies and agreement between D150 and FAV.

		Agreement		Accuracy	Prevalence		
150×	FAV	Gwet AC1	*p*	*p*	Prevalence 150×	Prevalence FAV	*p*
Keratinocytes	Small pigmented cells with sharp borders	0.86	<0.001	0.88	96.1%	93.7%	0.772
Roundish melanocytes	Large isolated cells with clearly visible sharp borders	0.70	<0.001	0.82	32.1%	25.4%	0.497
Dendritic melanocytes	Large isolated dendritic cells	0.50	0.001	0.68	19.2%	23.8%	0.650

## Data Availability

The data presented in this study are available on request from the corresponding author.
